# The effect of low resolution and coverage on the accuracy of susceptibility mapping

**DOI:** 10.1002/mrm.27542

**Published:** 2018-10-19

**Authors:** Anita Karsa, Shonit Punwani, Karin Shmueli

**Affiliations:** ^1^ Department of Medical Physics and Biomedical Engineering University College London London United Kingdom; ^2^ Centre for Medical Imaging University College London London United Kingdom

**Keywords:** decreased contrast, low resolution, magnetic susceptibility, magnetic susceptibility mapping, QSM accuracy, reduced field‐of‐view

## Abstract

**Purpose:**

Quantitative susceptibility mapping (QSM) has found increasing clinical applications. However, to reduce scan time, clinical acquisitions often use reduced resolution and coverage, particularly in the through‐slice dimension. The effect of these factors on QSM has begun to be assessed using only balloon phantoms and downsampled brain images. Here, we investigate the effects (and their sources) of low resolution or coverage on QSM using both simulated and acquired images.

**Methods:**

Brain images were acquired at 1 mm isotropic resolution and full brain coverage, and low resolution (up to 6 mm slice thickness) or coverage (down to 20 mm) in 5 healthy volunteers. Images at reduced resolution or coverage were also simulated in these volunteers and in a new, anthropomorphic, numerical phantom. Mean susceptibilities in 5 brain regions, including white matter, were investigated over varying resolution and coverage.

**Results:**

The susceptibility map contrast decreased with increasing slice thickness and spacing, and with decreasing coverage below ~40 mm for 2 different QSM pipelines. Our simulations showed that calculated susceptibility values were erroneous at low resolution or very low coverage, because of insufficient sampling and overattenuation of the susceptibility‐induced field perturbations. Susceptibility maps calculated from simulated and acquired images showed similar behavior.

**Conclusions:**

Both low resolution and low coverage lead to loss of contrast and errors in susceptibility maps. The widespread clinical practice of using low resolution and coverage does not provide accurate susceptibility maps. Simulations in images of healthy volunteers and in a new, anthropomorphic numerical phantom were able to accurately model low‐resolution and low‐coverage acquisitions.

## 1 INTRODUCTION

Quantitative susceptibility mapping (QSM) is an emerging MRI technique that can reveal disease‐related changes in tissue iron, myelin and calcium content, and venous oxygenation. Therefore, QSM shows potential for an increasing range of clinical applications.^1^ Magnetic susceptibility (χ) is an intrinsic tissue property relating the magnetic field induced within the tissue to the applied magnetic field. The relationship of $\chi \left(r\right)$, the tissue susceptibility distribution over space (r), to the resulting phase variations ($\varphi \left(r\right)$) can be expressed by the following convolution with the dipole field distribution ($d\left(r\right)$) where $B_{0}$ denotes the main magnetic field^2^, ^3^:

(1) $$\varphi \left(r\right)\propto B_{0}\left(\chi \left(r\right)*d\left(r\right)\right).$$

QSM recovers the inherent tissue susceptibility distribution from gradient‐echo phase images in 3 conceptual steps: phase unwrapping, background field removal, and susceptibility calculation.^4^, ^5^, ^6^, ^7^

One advantage of QSM is that it does not require any additional special hardware or sequences. In theory, susceptibility maps can be calculated from any MR image acquired with a T_2_^*^‐weighted gradient‐echo pulse sequence. Therefore, QSM can provide additional information for clinicians when gradient‐echo images are acquired as part of the diagnostic MRI protocol. However, clinical images are often acquired with large slice thickness^8^, ^9^, ^10^, ^11^, ^12^, ^13^, ^14^ and reduced coverage in the through‐slice dimension^8^, ^15^, ^16^, ^17^, ^18^, ^19^ to shorten scans and increase patient throughput. As QSM techniques are increasingly applied in clinical imaging, it is imperative to understand the effect of low resolution and coverage on susceptibility maps.

Recent studies have begun to investigate the effect of resolution on the accuracy of QSM. In Li et al. 20 MR images of 5 healthy volunteers acquired with 1 mm isotropic resolution were downsampled in the through‐slice dimension to simulate slice thicknesses of 2 and 4 mm, followed by QSM. The study found the error in susceptibility because of increased slice thickness to be negligible. However, Haacke et al. 4 found a 10–25% decrease in the susceptibility of iron‐rich deep‐brain structures (e.g., globus pallidus and caudate nucleus) when increasing the slice thickness from 0.5 to 3 mm in a numerical brain phantom. Zhou et al. 21 acquired images of gadolinium‐filled balloon phantoms at 4 different isotropic resolutions (0.7–1.8 mm) and reported a decrease in the calculated susceptibility with decreasing resolution. Sun et al. 22 acquired brain images of a healthy volunteer at different isotropic resolutions (2, 3, and 4 mm) using a sequence optimized for functional QSM. They reported a decrease in mean susceptibility in 5 iron‐rich brain regions with increasing voxel size. These findings indicate that, at least over a narrow resolution range (0.5–4 mm), there is a decrease in absolute susceptibility values with increasing voxel size.

One study has investigated the effect of spatial coverage on QSM. In Elkady et al. 23 5 healthy volunteers were scanned with a resolution of 0.5 × 0.75 × 2 mm and full coverage of the brain. Spatial coverage was incrementally truncated post‐acquisition to a minimum through‐slice FOV of 12 mm centered on the globus pallidus (GP). A susceptibility error of more than 5% was found in the GP for FOVs smaller than 5.6 times its size in the through‐slice dimension. This result implies that capturing the full extent of the susceptibility‐induced phase or field variations is necessary for accurate QSM.

Based on the results of these studies, we hypothesize that either low resolution or low coverage will result in reduced and erroneous absolute susceptibility values. We aimed to overcome the limitations of previous studies. For example, Haacke et al. 4 used a numerical brain phantom instead of acquired images, whereas Elkady et al. 23 simulated a decreased FOV by excluding slices post‐acquisition. Here, in addition to performing simulations in a numerical phantom, we also acquired MR images at different resolutions and FOVs in healthy volunteers.^24^, ^25^ Although Zhou et al. 21 did acquire images at different resolutions, balloon phantoms are not sufficient to model detailed human brain anatomy. Therefore, we collected in vivo brain images to show that this effect is substantial in healthy volunteers. Finally, the brain acquisitions of Sun et al. 22 used EPI and were tailored for functional QSM, whereas we used a 3D gradient‐echo sequence designed for structural QSM. We also used a broader range of slice thickness and coverage than any of the previous studies and explored the effect of slice spacing on QSM for the first time. Furthermore, we used 3D gradient‐echo imaging instead of a 2D multi‐slice sequence to acquire images at different resolutions as 3D imaging is becoming the sequence‐of‐choice for QSM. In addition, we included the white matter in the analysis instead of only investigating deep‐brain structures as in all previous studies. Moreover, we compared images acquired in healthy volunteers with downsampled images of the same volunteers to test the applicability of post‐acquisition downsampling. We also investigated the source of the error introduced by a reduced coverage by downsampling the acquired images at different stages of the QSM pipeline. Here, we created a new, high‐resolution, anthropomorphic, head‐and‐neck numerical phantom and used it to compare simulated and acquired images. Simulations performed on the numerical phantom also enabled us to compare calculated susceptibility values against ground‐truth values to allow quantitative measurement of QSM accuracy.

## 2 METHODS

### 2.1 Data acquisition

The local ethics committee approved this study and informed consent was obtained from all participants.

To investigate the effect of reduced through‐plane resolution (used in clinical practice) on QSM, multi‐echo brain images were acquired in 5 healthy female volunteers (age range: 26–30 years) at 3T (Achieva, Philips Healthcare, NL) using a 3D gradient‐echo pulse sequence tailored for structural QSM and a 32‐channel receiver head coil, with matrix size = 240 × 240 × 144, SENSE factors = 1 × 2 × 1.5, 1 mm in‐plane resolution, TE_1 _= 3 ms, ΔTE = 5.4 ms, 5 echoes, TR = 29 ms, flip angle = 20°, and slice thicknesses 1, 2, 4, and 6 mm. Multiple echoes were acquired rather than single echoes as these have been shown to provide more accurate field estimation.^4^, ^26^, ^27^

To investigate the effect of reduced through‐plane coverage (used in clinical practice) on QSM, the same volunteers were also scanned using a 2D gradient‐echo pulse sequence to avoid aliasing in the through‐slice (head‐foot) direction. A 2D acquisition was also most appropriate here as clinical studies often use 2D multi‐slice acquisitions. A 32‐channel receiver head coil was used with matrix size = 240 × 240 in‐plane, SENSE factors = 1 × 2, 1 mm isotropic resolution, TE_1_ = 4.9 ms, ΔTE = 5.3 ms, 5 echoes, TR = 4549 ms, flip angle = 90°, and a through‐slice FOV of 144, 111, 78, and 44 mm centered around the middle of the brain. Post‐acquisition downsampling in the first 2 volunteers predicted a sharp decrease in susceptibility contrast below a coverage of 44 mm. Therefore, the last 3 volunteers were also scanned with a 20‐mm through‐slice FOV.

The scanner‐reconstructed, post‐coil combination magnitude and phase images were used for all subsequent image processing in each case.

### 2.2 Designing a realistic numerical head‐and‐neck phantom

We compared the acquisitions with simulations performed on a numerical phantom to investigate whether the phantom could accurately model in vivo brain images and to assess how the estimated susceptibilities compared to ground‐truth values. A numerical phantom of 1 mm isotropic resolution was necessary to model the highest‐resolution acquisitions. To accurately model the background fields in the brain, the phantom needed to include the entire head‐and‐neck. Because no such numerical phantom was available, we created our own realistic, high‐resolution, numerical, head‐and‐neck phantom based on the Zubal phantom.^28^

**Figure 1:**
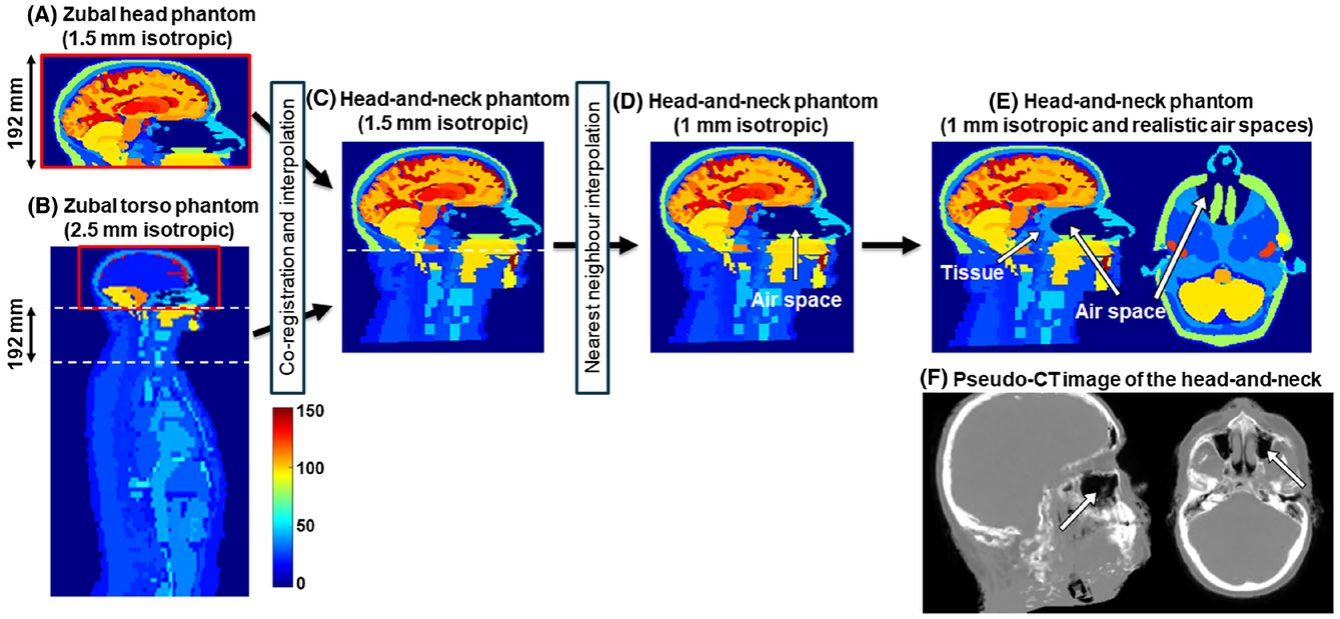
Creation of a realistic, numerical head‐and‐neck phantom. The Zubal head phantom (A) was modified to include the neck section (C) from the torso phantom (B), interpolated to achieve 1 mm isotropic resolution (D) and the oropharyngeal air space was made smaller and more realistic (E and F). The overlapping regions in (A) and (B) are outlined in red. Images (A)–(E) display the indices of different regions used in the original Zubal phantoms. Pseudo‐CT images (F) were generated from a proton density map of a healthy volunteer using an online pseudo‐CT synthesis tool^32^, ^33^, ^34^

The Zubal phantom is an anthropomorphic model of the human head and torso. It contains indexed regions designating anatomical structures in the brain and the body obtained by manual segmentation of (spoiled gradient‐echo) MRI and CT images of 2 human male volunteers. The head (Figure 1A) and the torso (Figure 1B) phantoms have initial isotropic resolutions of 1.5 mm and 2.5 mm, respectively.

A new, high‐resolution, anthropomorphic head‐and‐neck phantom was created in 3 steps (Figure 1):

1. The overlapping torso and head phantoms (index maps) were rigidly co‐registered in the overlapping region (outlined in red in Figure 1A and B) using the NiftyReg toolbox^29^ with default settings and nearest‐neighbor interpolation. The co‐registered and interpolated 192 mm neck section (Figure 1B) of the torso phantom was then attached to the head phantom (Figure 1C) to obtain a full head‐and‐neck phantom of 1.5 mm isotropic resolution and a matrix size of 256 × 256 × 256.
2. The resulting numerical head‐and‐neck phantom was further improved by nearest‐neighbor interpolation of the indices in MATLAB (The MathWorks, Natick, MA) to achieve 1 mm isotropic resolution (Figure 1D) and a matrix size of 384 × 384 × 384.
3. Preliminary simulations showed that the largest oropharyngeal air space in the numerical phantom (Figure 1D) induced strong background fields in the brain that proved to be very difficult to remove completely using standard methods such as projection onto dipole fields (PDF)^30^ or the Laplacian boundary value method (LBV).^31^ However, none of the acquired images contained such large background fields. Compared to a pseudo‐CT image of the head‐and‐neck (Figure 1F),^32^, ^33^, ^34^ the air space in the numerical phantom seemed too large with unrealistically sharp edges perpendicular to B_0_. Therefore, a simple ellipsoidal shape (center = [195, 61, 248], axes = [25, 65, 18] in voxel units) was used to make the oropharyngeal air space in the numerical phantom smaller and more anatomically realistic (Figure 1E).

**Figure 2:**
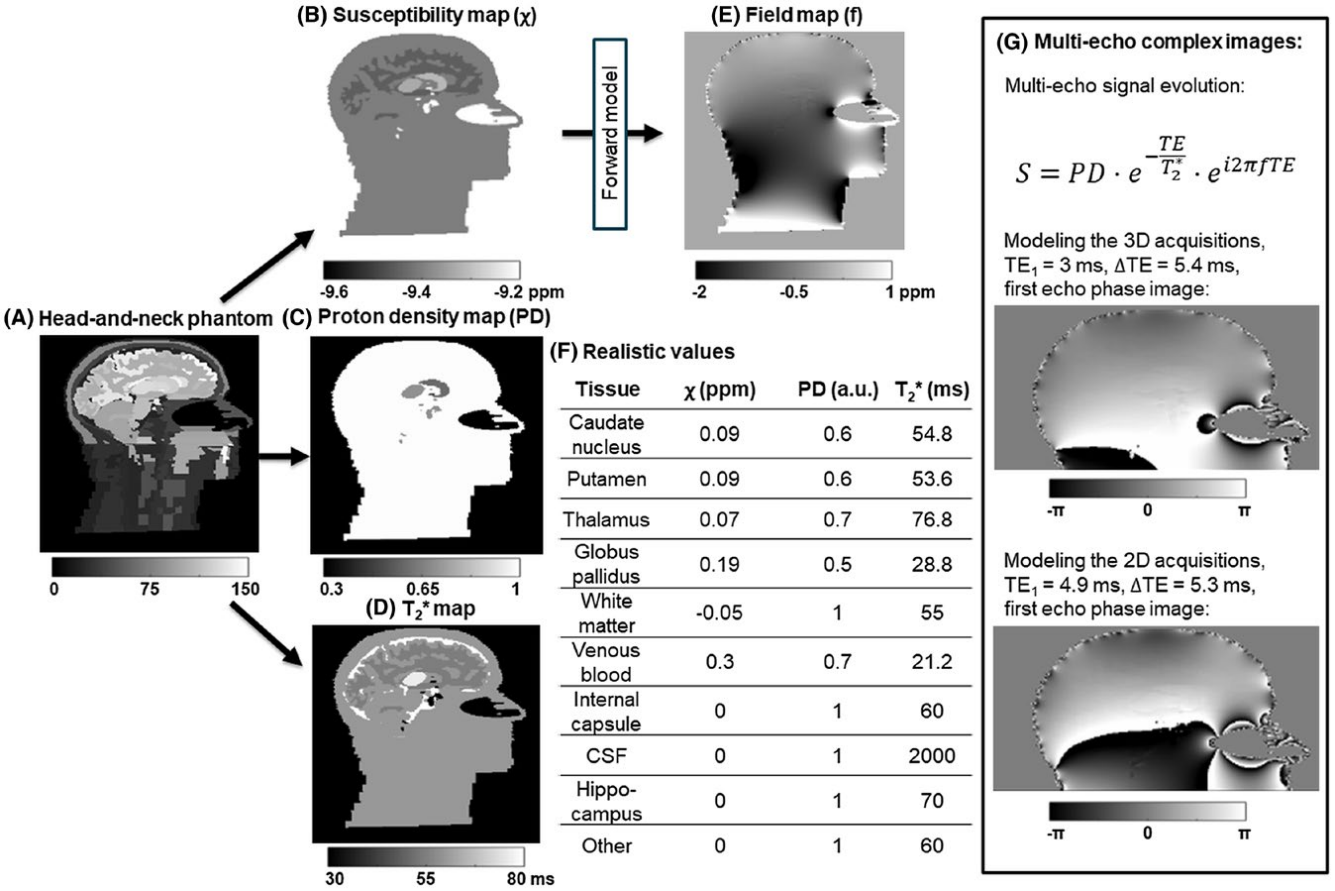
Multi‐echo brain images of the numerical phantom. Realistic susceptibility (B), proton density (C), and T_2_^*^ (D) values were assigned to several brain regions (F) based on previous literature.^35^, ^36^, ^37^, ^38^, ^39^ The field map (E) was calculated from the susceptibility map using a Fourier‐based forward model.^41^ Multi‐echo complex images (G) were calculated to model the 3D and 2D acquisitions

Realistic proton density, susceptibility and T_2_^*^ values, based on previous literature,^35^, ^36^, ^37^, ^38^, ^39^ (Figure 2F) were assigned to several brain regions in the numerical phantom (Figure 2B–D). The susceptibility values in Figure 2F are relative values referenced to −9.4 ppm (i.e., the susceptibility difference between tissue and air).^40^ A Fourier‐based forward model^41^ was applied to estimate a field map (Figure 2E) from the susceptibility distribution. Multi‐echo complex images (Figure 2G) were simulated in the head at 3T with:

- TE_1_ = 3 ms, ΔTE = 5.4 ms, 5 echoes and
- TE_1_ = 4.9 ms, ΔTE = 5.3 ms, 5 echoes

to model the 3D (varying resolution) and 2D (varying coverage) acquisitions, respectively.

### 2.3 Simulations

**Figure 3:**
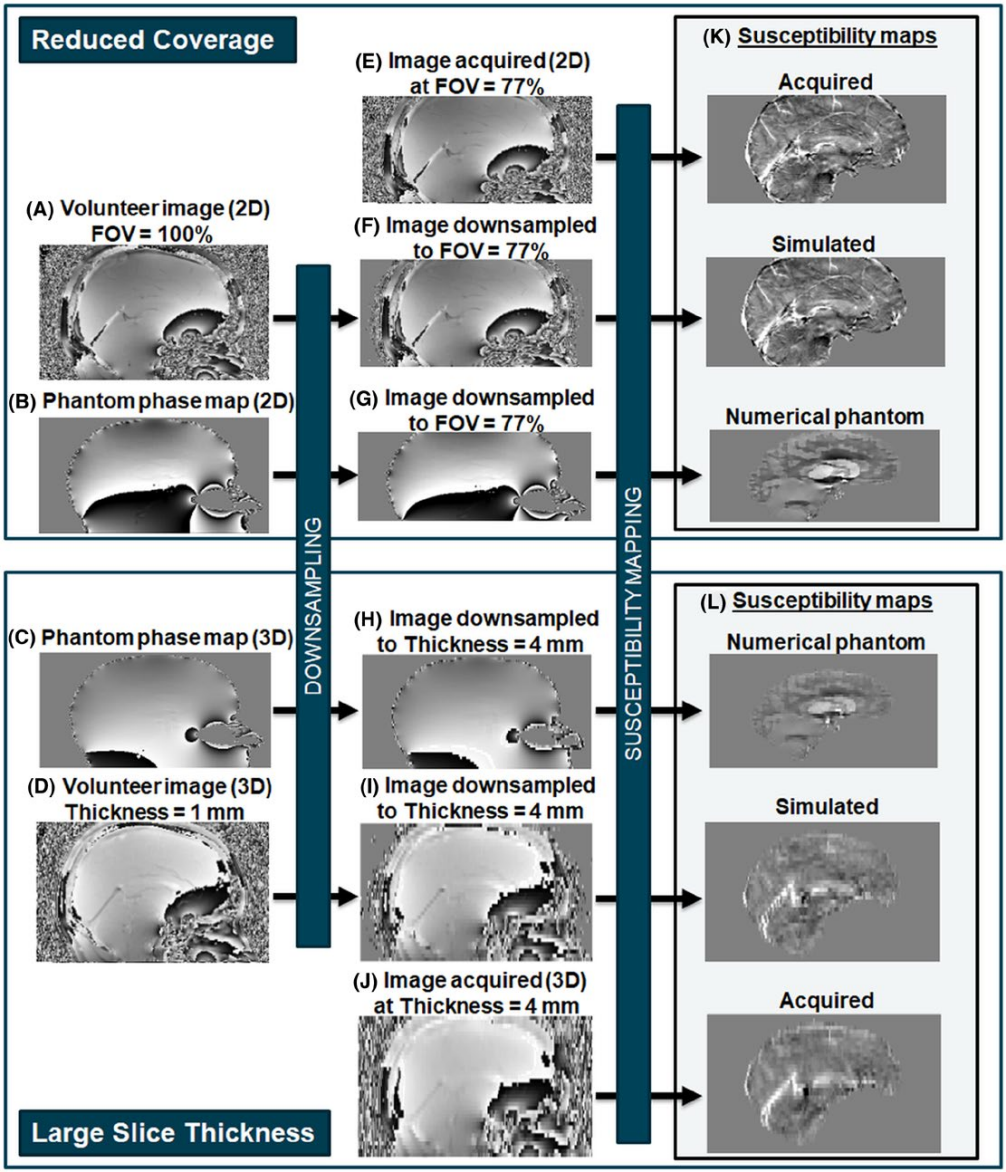
Flow chart to illustrate simulations of reduced coverage and resolution. Multi‐echo brain complex image volumes were simulated from a numerical phantom (B and C) and acquired in healthy volunteers (A and D). The effect of a reduced FOV (F and G) and reduced through‐slice resolution (H and I) were investigated by downsampling the images as well as acquiring low‐coverage (E) and low‐resolution (J) brain images. Susceptibility maps (K and L) were calculated and compared in each case

To compare the volunteer acquisitions with simulations in healthy volunteers and the numerical head‐and‐neck phantom, both high‐resolution, full‐coverage images of all 5 healthy volunteers and the complex simulated images of the numerical phantom were downsampled to simulate increased slice thicknesses or decreased FOVs (Figure 3).

Low‐resolution complex MRI images were simulated from the full‐resolution 3D volunteer acquisitions (Figure 3D) and the simulated multi‐echo numerical phantom images (Figure 3C) by averaging the complex image across each slab of *m* = 2–6 mm slices (Figure 3H and I).

Low‐coverage images were simulated from the full‐coverage 2D volunteer acquisitions (Figure 3A) and the simulated multi‐echo numerical phantom images (Figure 3B) by including only the central *N* = 89%, 77%, 66%, 54%, 21%, and 14% of the slices (Figure 3F and G). Full brain coverage corresponds to *N *= 100%.

Low‐resolution images with isotropic voxels but large slice spacing were also simulated in the numerical phantom by including only every *P* = 2nd to 6th slice in the initial complex image.

### 2.4 Susceptibility mapping (QSM) pipeline

Susceptibility maps were calculated from all complex images (Figure 3K and L) using the following susceptibility mapping pipeline:

1. non‐linear field fitting,^35^, ^42^
2. Laplacian phase unwrapping,^42^, ^43^
3. background field removal using PDF,^30^, ^42^ and
4. susceptibility calculation using truncated k‐space division (TKD^2^ with δ = 2/3 and correction for susceptibility underestimation using the point spread function from Schweser et al.)^43^

**Figure 4:**
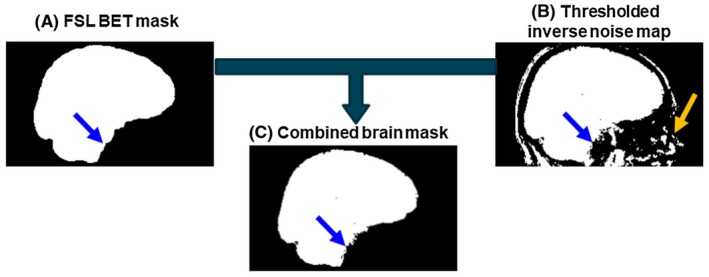
Brain mask creation. The final brain mask (C) is the intersection of an FSL BET mask (A) excluding all the tissue outside of the brain (orange arrow), and the thresholded inverse noise map (B) excluding all voxels with high noise (blue arrows)

Zero padding, to matrix sizes of 256 × 256 × 256 and 384 × 384 × 256 for the volunteer and numerical phantom images, respectively, was applied before steps 2 and 4. Brain masks were generated by combining (Figure 4C) the results of the FSL Brain Extraction Tool^44^ applied to the last‐echo magnitude image (Figure 4A) and thresholding of the inverse noise map^42^^,^ 45 (Figure 4B). The first mask excluded voxels outside the brain (Figure 4B, orange arrow), whereas the second mask ensured that only low‐noise regions were included (Figure 4, blue arrows) in the susceptibility calculation reducing streaking artefacts from noisy voxels at the brain edges.

It could be argued that direct k‐space inversion methods, such as TKD, might reconstruct inaccurate susceptibility maps at low resolution and coverage because they implicitly assume that the field values outside the tissue mask are zero instead of noisy or unknown. In theory, susceptibility calculation (SC) techniques that perform fitting of the field map within the tissue mask in image space could overcome this problem. To test whether errors in the estimated susceptibility could be corrected using an image‐space fitting technique, we applied the same QSM pipeline as described above but with morphology enabled dipole inversion (MEDI)^42^, ^46^ as the final step instead of TKD for all the acquired volunteer images and the numerical phantom.

To investigate the source of the error introduced by a reduced coverage, we performed the aforementioned post‐acquisition downsampling (from *N* = 100% to 14%) not only before the QSM pipeline, but after steps 2 or 3 as well. This was repeated with the above QSM pipeline but with LBV^31^ as the background field removal (BFR) step to examine how the results are affected by different BFR techniques. LBV was chosen because it was shown to perform similarly well to PDF in the brain while introducing slightly different error patterns near the tissue edges.^47^

### 2.5 Regions of interest in the brain

The mean and SD of susceptibilities in several brain regions were used to compare the acquisitions and simulations, because the mean is the summary susceptibility measure used in the majority of studies applying QSM. Only regions that have been widely studied in the susceptibility mapping community because of their paramagnetic or diamagnetic nature were included. Regions that were not part of the Zubal segmentation (e.g., red nuclei) were excluded. Therefore, the 5 regions of interest (ROIs) used in this study were caudate nucleus, putamen, thalamus, globus pallidus, and white matter.

Brain ROIs (shown in Supporting Information Figure S2A) in the full‐resolution, full‐coverage images were obtained via non‐rigid registration of the Eve atlas magnitude image^48^ and the last‐echo magnitude images because these had the most similar echo times and image contrast. The registration was performed using NiftyReg^29^ with 5 levels, a final grid spacing of 3 voxels, and the weight of the bending energy term set to 0.03. This provided suitable segmentations of the ROIs in all 5 healthy volunteers as assessed by visual inspection.^49^ The posterior corona radiata was used as a white matter ROI in the volunteer images, as unlike other ROIs in the Eve atlas (e.g., superior corona radiata), it never had an overlap with the gray matter or any of the iron‐rich deep‐brain regions. The posterior limb of the internal capsule was also segmented in the volunteer images, because it has been found to be the best reference tissue for susceptibility maps.^50^

To obtain the aforementioned ROIs for the low‐resolution and low‐coverage images, the full‐resolution, full‐coverage susceptibility maps were rigidly co‐registered with all other susceptibility maps in the same volunteer using MATLAB's imregister function. The resulting transformations were then applied to the ROIs in the full‐resolution, full‐coverage image using MATLAB’s imwarp function to obtain the same regions in the low‐resolution or low‐coverage images.

The mean and SD of susceptibilities were calculated in the 5 brain ROIs and referenced to the posterior limb of the internal capsule for the volunteers and the whole internal capsule for the numerical phantom as the latter does not have subsegments in the phantom. The average and SD of the mean susceptibility in each ROI were calculated across subjects. To show that our results did not originate from edge effects at very low coverage, the same analysis was repeated in the images acquired with the lowest coverage, but the mean was calculated in the middle slice only.

SNR was estimated in the first‐echo magnitude images acquired at the highest resolution and at full coverage. The ratio between the mean and SD of the signal magnitude in each ROI was used as a measure of SNR.

To compare our results with those of Elkady et al., 23 we estimated the coverage corresponding to a mean susceptibility in the GP that was 5% less than that in the full‐coverage case for each healthy volunteer. We used the results of the downsampling instead of the acquisitions as these were very similar, and we had more downsampled than acquired images. To determine the coverage corresponding to a 5% decrease (Cov_5%_) in the mean susceptibility of the GP, we estimated the mean susceptibility in the GP as a function of coverage using linear interpolation of susceptibilities between the simulated coverage values. The size of the GP in the through‐slice dimension for each healthy volunteer was also measured. We repeated this process for the rest of the ROIs.

## 3 RESULTS

### 3.1 Comparison of acquisitions in volunteers and simulations

**Figure 5:**
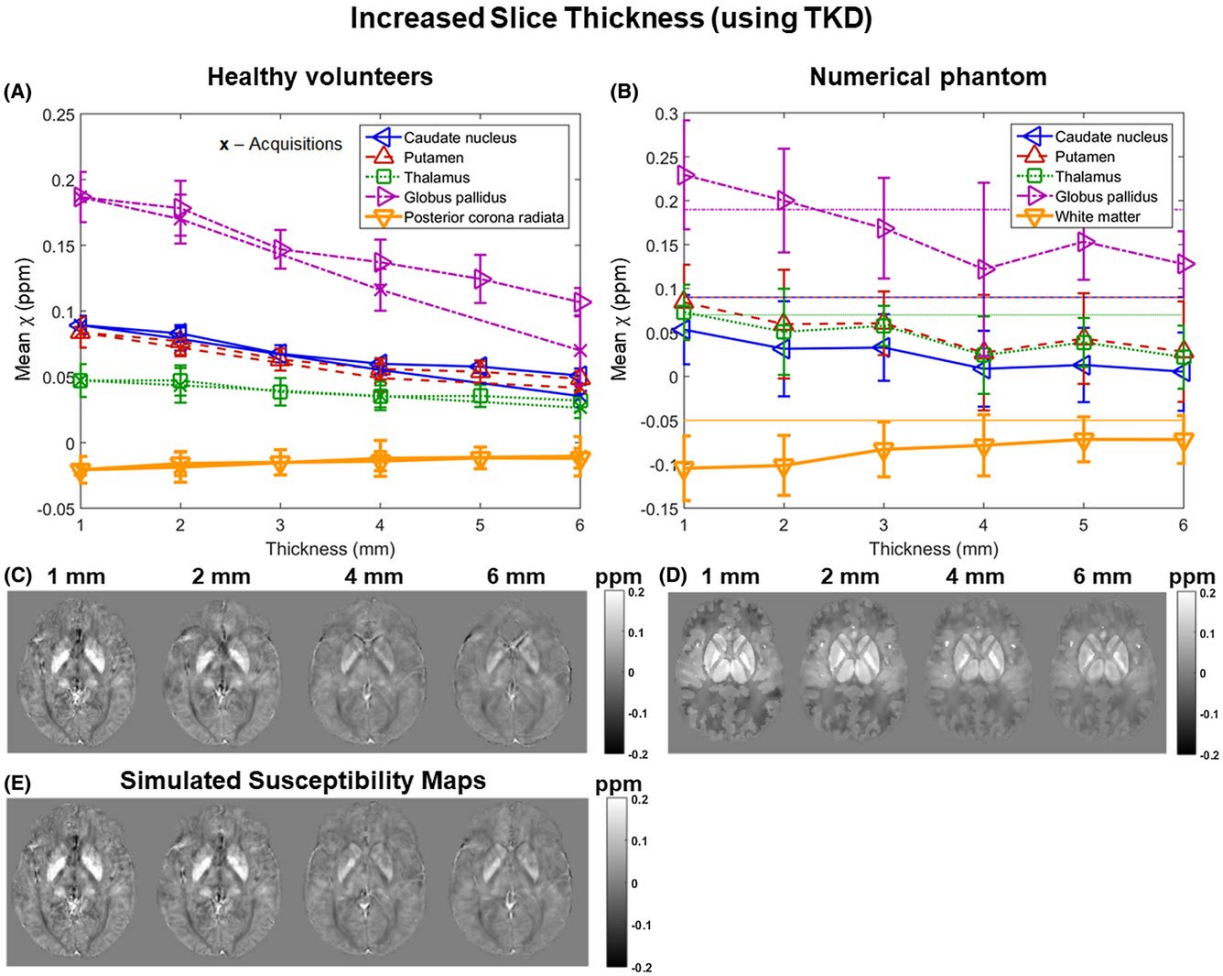
Susceptibility over varying slice thickness. Mean susceptibilities in 5 ROIs are plotted against slice thickness. Susceptibilities were averaged across 5 volunteers (A: x, acquisitions; triangles and square, simulations) and simulated in the numerical phantom (B: horizontal lines indicate the corresponding ground‐truth susceptibility values). The data acquired in volunteers (A) have error bars equal to the SD of the mean ROI values across the 5 volunteers. The error bars in the phantom results (B) correspond to the SD within the ROIs. Axial slices of susceptibility maps calculated from images at different slice thicknesses acquired (C) or simulated (E) in a representative volunteer and simulated in the numerical phantom (D) are also shown

**Figure 6:**
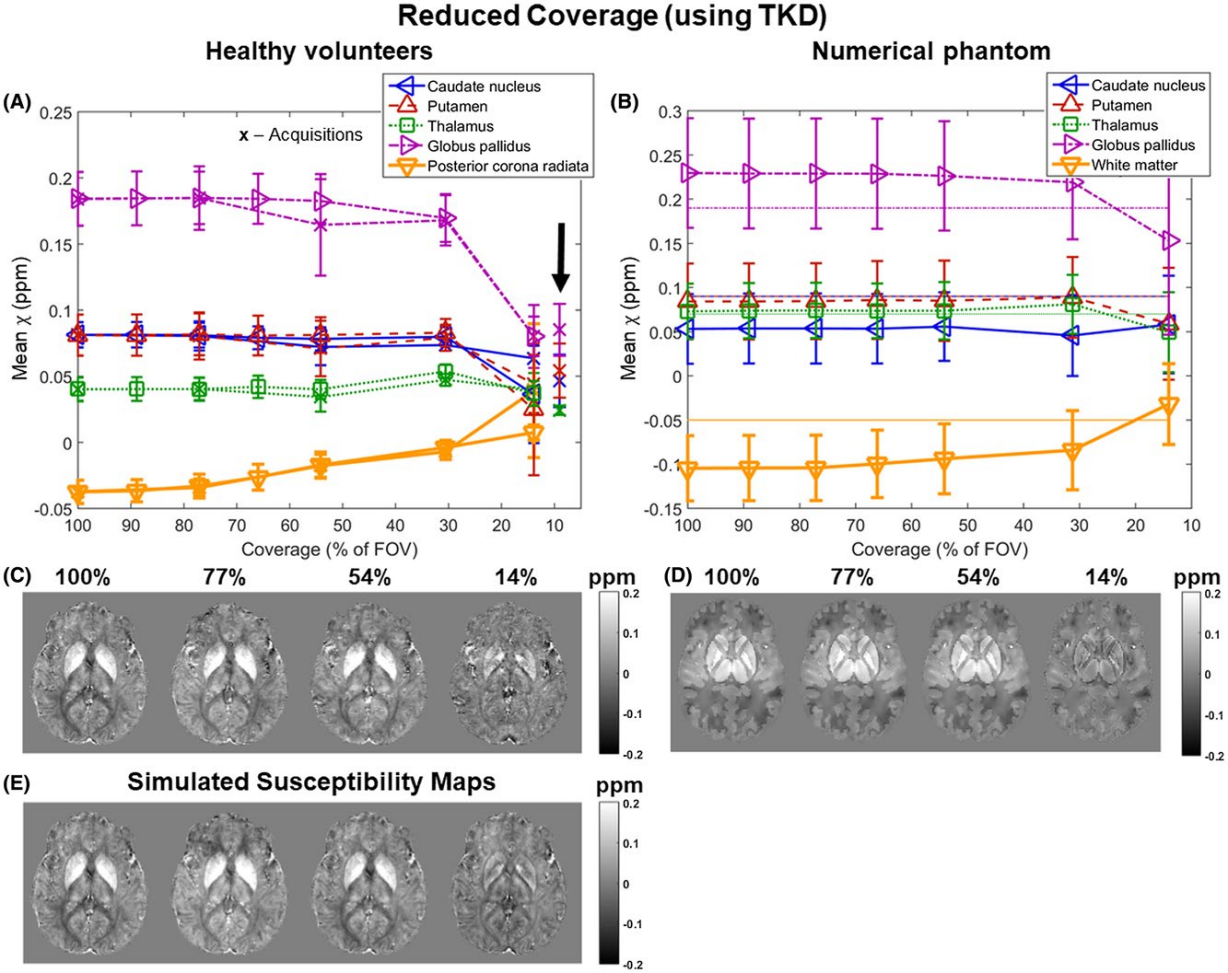
Susceptibility over varying coverage. Mean susceptibilities in 5 ROIs are plotted against coverage. Susceptibilities were averaged across 5 volunteers (A: x, acquisitions; triangles and square, simulations) and simulated in the numerical phantom (B: horizontal lines indicate the corresponding ground‐truth susceptibility values). The arrow (A) indicates mean susceptibilities in the ROIs calculated in the middle slice only in case of the lowest coverage acquisitions. The data acquired in volunteers (A) have error bars equal to the SD of the mean ROI values across the 5 volunteers. The error bars in the phantom results (B) correspond to the standard deviation within the ROIs. Axial slices of susceptibility maps calculated from images at different coverage acquired (C) or simulated (E) in a representative volunteer and simulated in the numerical phantom (D) are also shown

Figures 5 and 6 show the mean susceptibility in several brain regions over varying slice thickness and coverage respectively for the volunteers (Figures 5A and 6A, both acquisitions [x] and simulations [triangles and square]) and the numerical phantom (Figures 5B and 6B). For the numerical phantom, the comparison between the effect of increasing slice thickness and slice spacing is shown in Supporting Information Figure S1. Simulated susceptibility maps in both the volunteers (Figures 5E and 6E) and the numerical phantom (Figures 5D and 6D) showed similar behavior to maps from acquired images (Figures 5C and 6C).

In all cases, the susceptibility contrast between the 5 ROIs decreased with increasing slice thickness (Figure 5). The numerical phantom simulations show that increased slice thickness also led to inaccurate susceptibility values (Figure 5B where the ground‐truth values are indicated by the horizontal lines). In some cases, anisotropic voxels provided results which were numerically closer to the ground‐truth (e.g., white matter in Figure 5B). The trends and the susceptibility maps were very similar for the slice thickness and slice spacing simulations. The SNR in the first‐echo magnitude images acquired at the highest resolution was 17 ± 4 on average across all volunteers and ROIs.

Figure 6 shows that the mean susceptibilities in the small deep‐brain structures were roughly constant until the through‐slice coverage reached 30% of the full FOV (44 mm), below which the susceptibility contrast between the 5 ROIs dropped drastically. However, in the white matter, the decrease in contrast and absolute mean susceptibility started at ~75% of the full FOV in both the volunteer images and the numerical phantom (Figure 6A and B, orange lines). Again, the estimated susceptibility in some regions was closer to the ground‐truth at the lowest coverage (e.g., globus pallidus and white matter in Figure 6B). Mean susceptibilities calculated in the entire ROIs were very similar to those calculated in the middle slice only (Figure 6A, arrow) in the images acquired with the lowest coverage.

On average, a coverage of at least 4.2 ± 0.6 times the size of the globus pallidus was needed for its mean susceptibility to be within 5% of that at full coverage (see Supporting Information Figure S2B for a subject‐by‐subject breakdown). The same measure for the rest of the ROIs was: 2.1 ± 0.8 (caudate nucleus), 2.3 ± 0.8 (putamen), 1.2 ± 0.3 (thalamus), and 5.8 ± 0.4 (white matter). The SNR in the first‐echo magnitude images acquired at full coverage was 18 ± 4 on average across all volunteers and ROIs.

### 3.2 Results of different susceptibility calculation techniques

**Figure 7:**
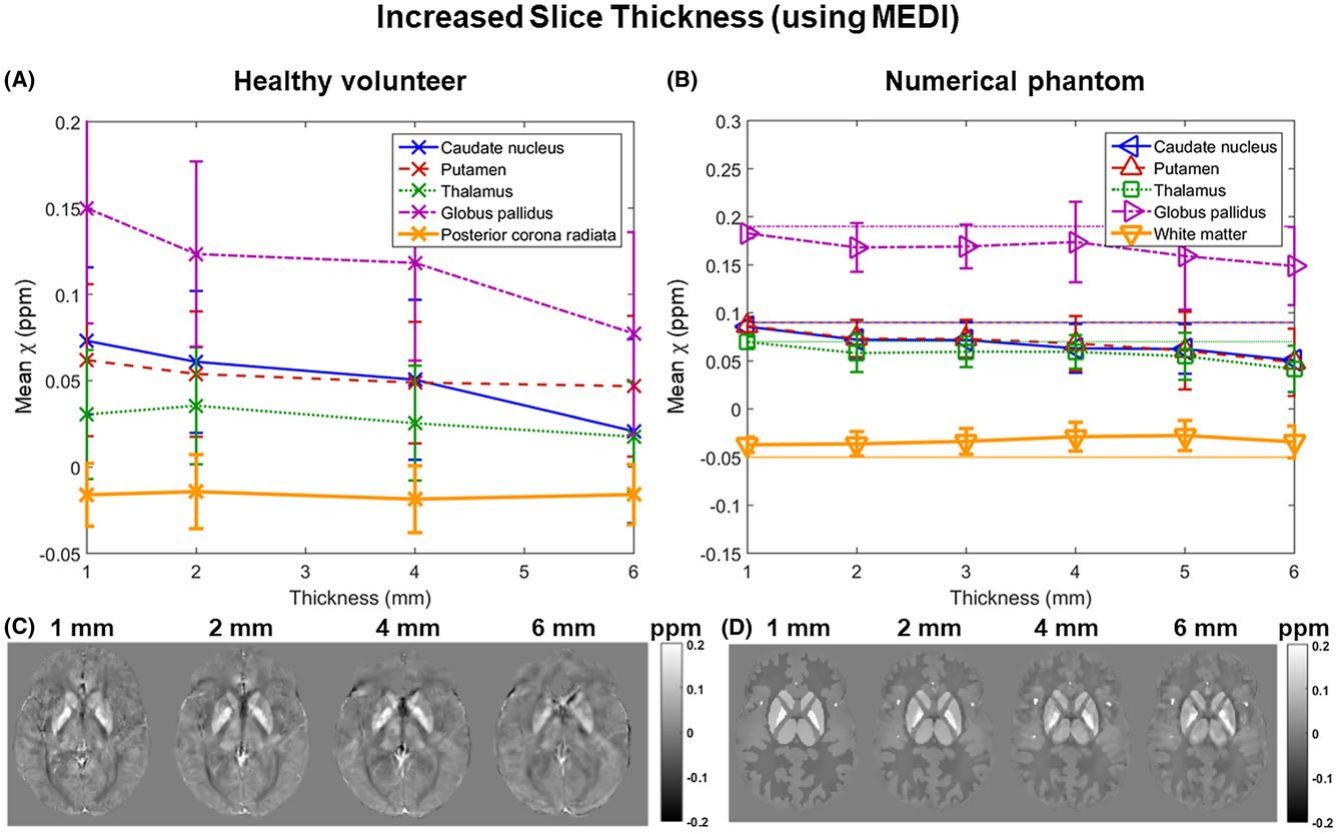
Susceptibility over varying slice thickness using MEDI instead of TKD for susceptibility calculation. Mean susceptibilities in 5 ROIs are plotted against slice thickness in a single, representative volunteer (A) and the numerical phantom (B). Note that here (unlike in Figures 5 and 6), the error bars in both graphs are equal to the SD within the ROIs. Axial slices of susceptibility maps from images at different slice thicknesses (C and D) are also shown

**Figure 8:**
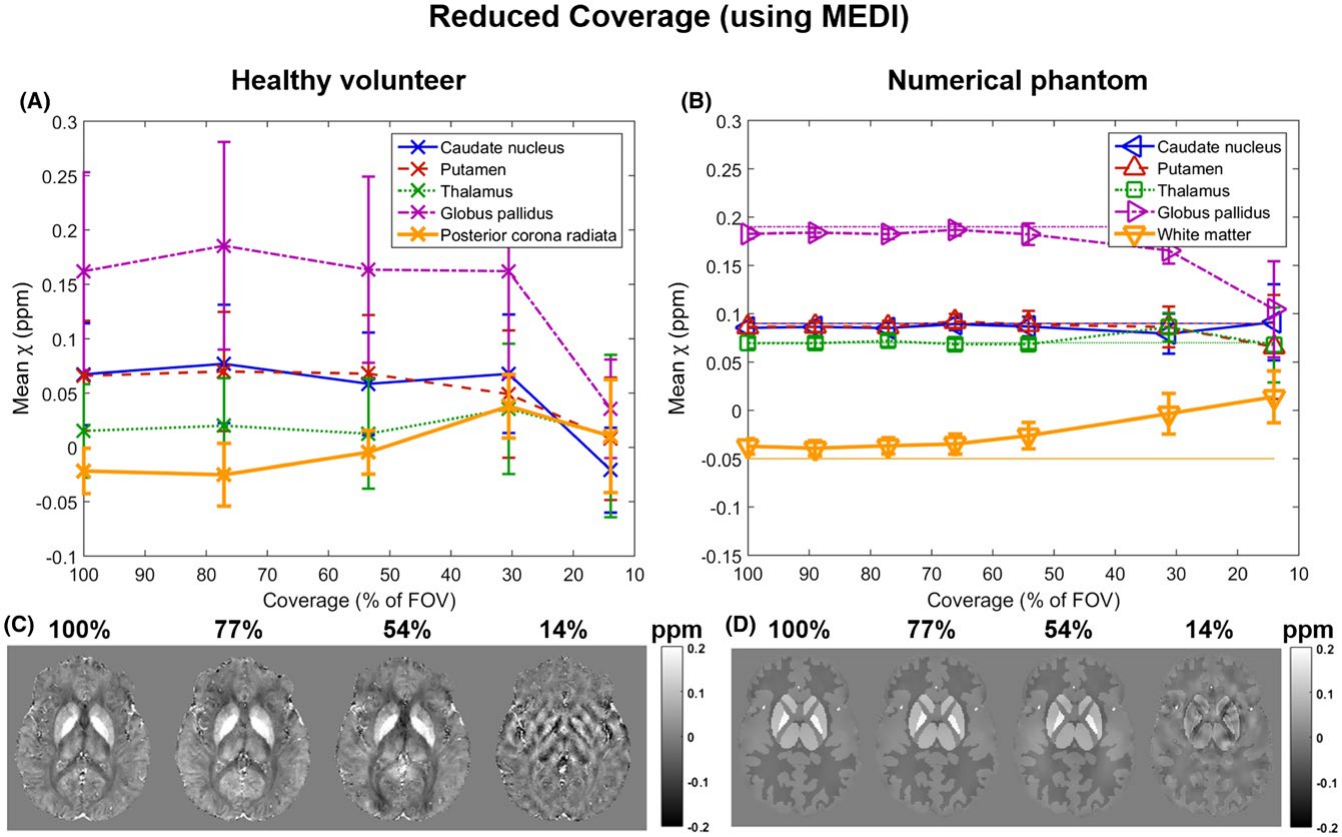
Susceptibility over varying coverage using MEDI instead of TKD for susceptibility calculation. Mean susceptibilities in 5 ROIs are plotted against coverage in a single, representative volunteer (A) and the numerical phantom (B). Note that here (unlike in Figures 5 and 6) the error bars in both graphs are equal to the SD in the ROIs. Axial slices of susceptibility maps from images at different FOVs (C and D) are also shown

Figures 7 and 8 show the mean susceptibility in 5 ROIs over varying slice thickness and coverage respectively calculated using MEDI in a single representative volunteer (Figures 7A, 7C, 8A, and 8C) and the numerical phantom (Figures 7B, 7D, 8B, and 8D). The results were very similar for all volunteers (not shown). In the volunteer images (Figures 7A, 7C, 8A, and 8C), the trends and susceptibility maps look similar to those in Figure 5 for increasing slice thickness (Figure 7) and in Figure 6 for decreasing coverage (Figure 8). Note that Figures 5A and 6A show mean susceptibilities across subjects, whereas Figures 7A and 8A show results in a single subject. For the single‐subject comparison between MEDI and TKD, see Supporting Information Figures S3 and S4.

In the numerical phantom (Figures 7 and 8, B and D), the results suggest that the MEDI algorithm was able to reproduce the susceptibility distribution at full resolution and coverage (Figures 7B and 8B) more accurately than TKD (Figures 5B and 6B). Furthermore, the MEDI pipeline seems to be more robust against increased slice thickness (Figures 5B and 7B) when applied to the numerical phantom even though a slight loss of contrast was still present at lower resolutions. However, TKD was more robust to decreased coverage (Figures 6B and 8B).

In general, the MEDI results had slightly lower contrast than the TKD results in the high resolution case and, consequently, their decrease in susceptibility contrast at large slice thickness was less pronounced.

Note that the error bars now correspond to the SD within each ROI. These are generally smaller in the numerical phantom than in the volunteer (Supporting Information Figures S3 and S4) especially when MEDI was applied to the numerical phantom.

### 3.3 The source of the error introduced by a very low coverage

**Figure 9:**
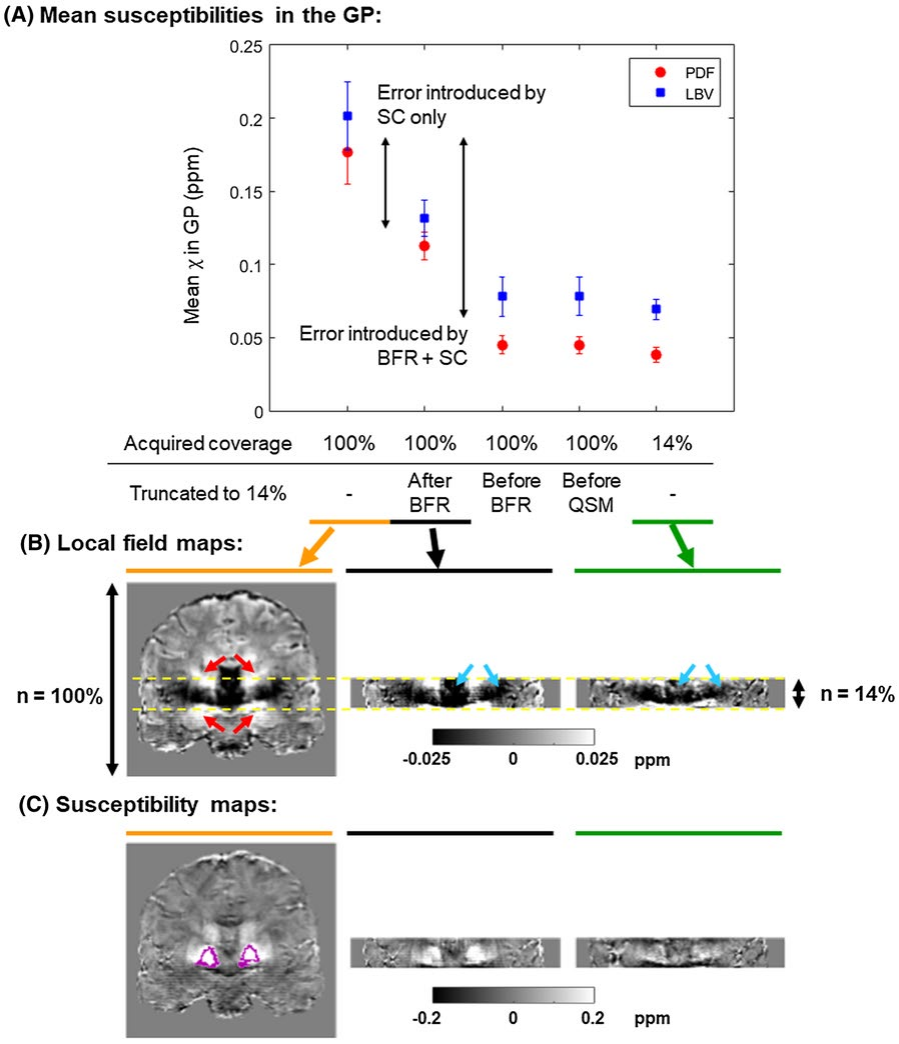
Investigating the source of the error introduced by a reduced coverage. Mean susceptibilities in the GP calculated in images acquired at *N* = 100%, downsampled from *N *= 100% to 14% after BFR, before BFR, or before QSM, and acquired at *N* = 14% are shown (A). The error bars correspond to the SD across subjects. The same experiment was performed using 2 different BFR techniques (PDF and LBV). The double arrows indicate the amount of error introduced by BFR + SC and SC only. Coronal slices of the local field (B) and susceptibility (C) maps calculated as part of the QSM pipeline (after step 3 and step 4, respectively) are shown in a representative healthy volunteer acquired with FOVs 100% (left), 14% (right), and downsampled after BFR (middle). The GPs are outlined in pink. Regions of the induced field perturbations (indicated by red arrows) are cut off at 14% coverage. Moreover, the field variations are attenuated near the volume edges in the image acquired at *N* = 14% (blue arrows). These factors seem to be the primary sources of the reduced susceptibility contrast at low coverage

Figure 9A shows mean susceptibilities calculated in the GP in the images acquired at full coverage, downsampled from *N* = 100% to 14% after BFR, before BFR, or before applying the QSM pipeline, and acquired at 14%. Although the susceptibilities provided by the LBV pipeline were consistently higher, both pipelines showed similar trends. The susceptibilities calculated from the images acquired at *N * = 14% or downsampled before BFR or QSM were very similar, whereas the results obtained by downsampling after BFR were halfway between the susceptibilities from images acquired at *N* = 100% or 14%. The double arrows indicate the error induced by BFR + SC and SC only. Example local field and susceptibility maps are also shown (Figures 9B and C, respectively).

## 4 DISCUSSION

We compared mean susceptibilities in 5 ROIs over varying slice thickness, slice spacing, and coverage. Susceptibility maps were calculated from images acquired in 5 healthy volunteers, downsampled images of the same volunteers, and images simulated in a numerical phantom designed to mimic detailed human brain anatomy. We also compared the variation of mean susceptibilities over slice thickness and coverage using 2 fundamentally different susceptibility calculation (SC) techniques (TKD and MEDI) and investigated the source of the error introduced by a reduced FOV using 2 state‐of‐the‐art background field removal (BFR) methods (PDF and LBV). Increased slice thickness and slice spacing as well as very low coverage all resulted in loss of contrast in the susceptibility maps. The error at very low coverage is introduced at the BFR and SC steps. The trends in mean susceptibilities were very similar for the acquired and downsampled volunteer images and for the numerical phantom. Susceptibility maps obtained using the 2 different pipelines were also very similar in the images acquired in volunteers.

The ranges of susceptibilities for each ROI according to previous literature^51^, ^52^, ^53^, ^54^, ^55^ are caudate nucleus (0.04–0.11 ppm), putamen (0.04–0.13 ppm), thalamus (‒0.02–0.05 ppm), globus pallidus (0.12–0.21 ppm), and white matter (‒0.06–0.03 ppm). The susceptibility values measured in this work at high resolution and full coverage are all within these ranges. Moreover, the low SD of the susceptibilities across volunteers confirms the reproducibility of QSM in the brain.^56^, ^57^, ^58^

The results of the slice thickness experiments are in good agreement with the findings of Haacke et al., 4 Zhou et al., 21 and Sun et al. 22 who all reported decreasing absolute susceptibility with decreasing resolution for a numerical brain phantom, a balloon phantom, and brain acquisitions tailored for functional imaging, respectively. However, our results include a larger range of resolutions, volunteer acquisitions tailored for structural imaging, as well as downsampled volunteer images and simulations using a numerical phantom. Furthermore, we also simulated increasing slice spacing in the numerical phantom. This showed a decrease in susceptibility contrast with increasing slice spacing similar to that seen with increasing slice thickness. This reduced contrast was probably caused by insufficient sampling of the susceptibility‐induced field perturbations. As the slice thickness increased, the complex MRI signal was averaged across increasingly anisotropic voxels and information about the field variations was lost. This is the voxel sensitivity function (VSF) mixing effect described by Zhou et al. 21 reported that the mixing effect at lower resolutions reduces phase contrast (i.e., attenuates the susceptibility‐induced field variations) leading to a decrease in absolute susceptibility at lower resolutions. This means that the susceptibility of paramagnetic sources inducing positive field perturbations (such as the deep‐brain nuclei) decreases, whereas the susceptibility of diamagnetic sources inducing negative field perturbations (such as white matter) increases with decreasing resolution. Noise‐related effects are unlikely to contribute in our case because of the high SNR (around 17 even at the highest resolution).

In Elkady et al. 23 found a 5% error in the susceptibility of the GP, compared to the susceptibility calculated at full‐coverage, when the FOV decreased below 5.6 times the size of the GP which is higher than our result for a similar susceptibility error (i.e., Cov_5%_ = 4.2 ± 0.6). However, they also mention that the BFR technique they used eroded the brain by the kernel radius. Accounting for this renders their actual Cov_5%_ to ~4.7 that is comparable with our findings, even though we used a different QSM pipeline and did not center our FOV around the GP. In a study focusing on 1 deep‐brain structure, the FOV is expected to be centered around the ROI. Therefore, reducing coverage symmetrically from the top and bottom of the brain is a limitation of this study. In this way, however, we could include more regions in our analysis. In any case, the ROIs used in this study are very close to the center of the brain in the head–foot direction in our images (except the white matter region in the volunteers). Our results investigating the source of the errors caused by very low coverage indicates that the BFR and SC steps introduce about the same amount of error (Figure 9A, double arrows). BFR attenuates the local field components close to the mask edges (Figure 9B, blue arrows). More accurate BFR could potentially overcome this problem. Here, however, we used 2 state‐of‐the‐art techniques with similar results, and it has been shown that all known BFR techniques lead to errors toward the mask edges.^47^ The error introduced by the SC step is probably because of regions of the local field map being cut off at reduced coverage (Figure 9B, red arrows). We have shown that neither TKD nor MEDI could fully recover the original susceptibility contrast (Figure 8A–D). Both of these effects arise when the FOV is comparable to the extent of the susceptibility‐induced field variations. Therefore, the small deep brain regions are only affected by a very low coverage, while the decrease in absolute mean susceptibility in the white matter region started at around 75% of the full FOV as it is a much larger ROI with more extensive field variations in the numerical phantom, and it is off‐center in the volunteer images. The induced field variations that are attenuated or cut off are positive for paramagnetic (deep‐brain nuclei) and negative for diamagnetic (white matter) susceptibility sources, therefore, similarly to decreasing resolution, the absolute susceptibility decreases with decreasing through‐plane coverage.

The numerical phantom experiments suggest that the susceptibility of some ROIs (e.g., white matter) were closer to the ground‐truth at the lowest resolution or coverage (Figures 5B and 6B). However, this is because of the fact that the susceptibility mapping pipeline (TKD) is imperfect and overestimated absolute susceptibility values (i.e., produced higher susceptibility for paramagnetic sources and lower susceptibility for diamagnetic sources than their ground truth susceptibility values) in some regions in the high‐resolution, full‐coverage images. Therefore, the mean susceptibilities in these regions became numerically closer to the ground‐truth as the absolute susceptibility values decreased because of decreasing resolution or coverage. This effect is the reason for using mean susceptibilities in the ROIs instead of an error metric such as the RMS error that can be misleading when a solution with more artifacts or lower quality happens to be numerically closer to the ground‐truth. Note that this overestimation of the absolute susceptibility had a substantial effect at full resolution and coverage on the globus pallidus and white matter, but not on the caudate nucleus, putamen, and thalamus. This could be associated with the large size of the white matter and the high susceptibility of the globus pallidus in the phantom, but investigating this effect further is beyond the scope of this study. There is no known SC method that can reconstruct susceptibility maps completely accurately in an entire brain in vivo.^59^ Different susceptibility mapping pipelines over‐ or underestimate the susceptibilities of certain regions to variable extent. However, our experimental scheme aims to investigate the trends (i.e., to characterize the errors introduced by low resolution and coverage by varying only these 2 parameters).

In all cases, susceptibility maps calculated from downsampled images were very similar to maps calculated from images acquired with reduced resolution or coverage. This shows that downsampling of high‐resolution and full‐coverage MRI images modeled low‐resolution and low‐coverage acquisitions well. Moreover, simulations performed in our realistic, noise‐free numerical phantom also yielded similar results to those in volunteers. This supports the hypothesis that the errors in the estimated susceptibility are primarily introduced by low resolution or coverage rather than by noise, microstructural, hardware‐related, or physiological effects that were not present in the numerical phantom. A notable example of this is the susceptibility anisotropy of white matter that has been shown to affect its measured susceptibility.^60^ However, in this study the decreasing susceptibility contrast is similar for the volunteer (anisotropic white matter) and numerical phantom (isotropic white matter by design) images implying that susceptibility anisotropy does not contribute substantially to the error induced by low resolution and coverage.

The results of comparing the 2 different SC techniques suggest that MEDI could reconstruct the susceptibility maps in the numerical phantom with lower error than TKD and did not overestimate the susceptibilities of the ROIs. The numerical susceptibility phantom used in this study consists of distinct regions with piecewise constant susceptibilities. The regularization term of MEDI penalizes regions with high susceptibility gradients, therefore it is better‐suited to calculate piece‐wise constant susceptibility maps where gradients are small within the regions. This is reflected in the fact that it provided more accurate susceptibility maps than TKD in the numerical phantom, although this same property of MEDI might lead to oversmoothing in in vivo susceptibility maps. However, the fact that the volunteer results were very similar for the 2 techniques in both the slice thickness and the coverage experiments suggests that the numerical phantom simulations underestimated the errors introduced by MEDI. Susceptibility varies on a microstructural scale in vivo^60^, ^61^, ^62^ that is also confirmed by the lower SDs within each ROI in the numerical phantom compared to volunteer images. In conclusion, the numerical phantom might not be suitable for investigating the effect of resolution and coverage on QSM when the MEDI pipeline is used. However, the fact that the susceptibility maps calculated using the 2 pipelines were very similar in the healthy volunteer suggests that even iterative fitting methods, such as MEDI, cannot recover the loss of susceptibility contrast caused by low resolution and coverage. Note that MEDI provided susceptibility maps with a slightly narrower dynamic range than TKD at full resolution and coverage in vivo. This is probably caused by the susceptibility overestimation of TKD at full resolution and coverage together with the oversmoothing of MEDI.

It should be noted that our results are only applicable to brain studies. Although we gave a general qualitative explanation for the sources of the decreased contrast, it is possible that motion and fat–water chemical shift effects are the dominant factors in other parts of the body making the influence of resolution and coverage less pronounced.

**Figure 10:**
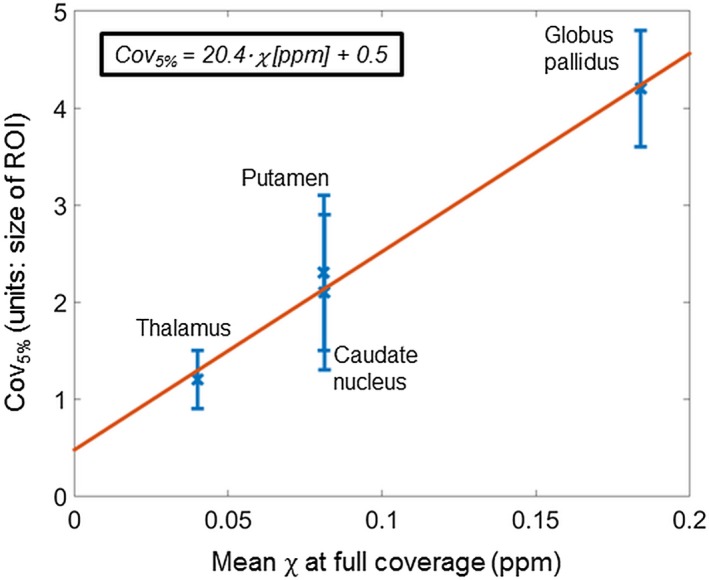
Linear relationship between the coverage necessary for a <5% decrease in susceptibility (Cov_5%_) and the mean susceptibility in 4 ROIs. The white matter region was excluded because of its high Cov_5%_ (5.8 ± 0.4). This high value was caused by its off‐center location that means that even a slightly reduced coverage affected its induced fields. The other 4 ROIs were all very close to the middle in the head‐foot direction. The least squares linear equation is displayed in the top left corner

In general, high resolution and large coverage provide more accurate susceptibility values. The fact that multiple studies using a variety of different QSM pipelines reported similar conclusions underlines the fact that currently the best way to avoid loss of susceptibility contrast is to carefully optimize these parameters. However, resolution and coverage are often constrained by the total scan time available. Moreover, very high resolution images acquired with a longer scan time are more likely to be corrupted by motion artifacts which could also introduce errors in the estimated susceptibility. Here, we have shown that for accurate susceptibilities, high resolution is necessary, and the FOV needs to exceed the structures of interest by a few times their size. The widespread clinical practice of imaging with large slice thickness, low coverage, and gaps between slices leads to loss of contrast and is not suitable for accurate QSM. The decrease in contrast may depend on various parameters such as the size, susceptibility, and shape of the ROI as well as its location within the FOV. Our crude estimations imply a linear relationship between the susceptibility of an ROI and its corresponding Cov_5%_ (Figure 10) that is in accordance with the fact that sources of higher susceptibility induce more extensive field variations. More ROIs and a finer grid of simulated resolutions and coverages would be necessary to determine an over‐arching relationship between the properties (e.g., size and susceptibility) of a susceptibility source, and the optimal resolution and coverage. Performing such experiments might be feasible using downsampling of in vivo, complex images, or simulations in our numerical phantom as we have shown that these model MRI acquisitions well. It might also be valuable to investigate the effect of low resolution and coverage in‐plane. Finally, future studies could explore the trends in susceptibility accuracy at even higher resolutions where the reduced SNR because of small voxels, motion issues because of long scan time, and microstructural effects are likely to influence and dominate the results.

## 5 CONCLUSIONS

Low resolution and low coverage lead to loss of contrast and errors in susceptibility maps. The widespread clinical practice of imaging at low resolution and coverage is not suitable for accurate susceptibility mapping. The reduced accuracy is probably caused by insufficient sampling (i.e., VSF mixing effect and cut‐off) and overattenuation (during background field removal near the mask edges) of the susceptibility‐induced local field variations. Based on this and similar studies so far, carefully optimized acquisitions seem to be the best solution to this problem. Determining the optimal parameters could be feasible using simulations that were shown to model the acquired images well.

## Supporting information

Figure S1 Susceptibility over varying slice thickness and slice spacing in the numerical phantom. Mean susceptibilities in 5 ROIs are plotted against slice thickness (A) and slice spacing (B) Horizontal lines indicate the corresponding ground‐truth susceptibility values in both graphs. The data acquired have error bars equal to the SD within the ROIs. Axial slices of susceptibility maps calculated from images at different slice thicknesses (C) and slice spacings (D) are also shown
Figure S2 (A) Regions of interest are shown on full‐coverage susceptibility maps in a representative healthy volunteer and the numerical phantom. The white matter region of the healthy volunteer (posterior corona radiata) is not shown as it is not in this slice. (B) The coverage necessary for <5% decrease (Cov_5%_) in the susceptibility of the globus pallidus is shown in each healthy volunteer
Figure S3 Susceptibility over varying slice thickness for different susceptibility calculation methods. Mean susceptibilities in 5 ROIs are plotted against slice thickness in a representative volunteer (A and D) and the numerical phantom (E and H) calculated using TKD (A, B, E, and F) or MEDI (C, D, G, and H). Note that here (unlike in Figures 5 and 6), the error bars in all graphs are equal to the SD within the ROIs. Axial slices of susceptibility maps from images at different slice thicknesses (B, C, F, and G) are also shown
Figure S4 Susceptibility over varying coverage for different susceptibility calculation methods. Mean susceptibilities in 5 ROIs are plotted against coverage in a representative volunteer (A and D) and the numerical phantom (E and H) calculated using TKD (A, B, E, and F) or MEDI (C, D, G, and H). Note that here (unlike in Figures 5 and 6), the error bars in all graphs are equal to the SD in the ROIs. Axial slices of susceptibility maps from images at different FOVs (B, C, F, and G) are also shown
