# Supplementary material for: The effect of low resolution and coverage on the accuracy of susceptibility mapping
Source: Magn Reson Med. 2018 Oct 19;81(3):1833–48. doi: 10.1002/mrm.27542 (PMC6492151; doi:10.1002/mrm.27542)
Supplement: Supplementary file 1 — Figure S1 Susceptibility over varying slice thickness and slice spacing in the numerical phantom. Mean susceptibilities in 5 ROIs are plotted against slice thickness (A) and slice spacing (B) Horizontal lines indicate the corresponding ground‐truth susceptibility values in both graphs. The data acquired have error bars equal to the SD within the ROIs. Axial slices of susceptibility maps calculated from images at different slice thicknesses (C) and slice spacings (D) are also shown Figure S2 (A) Regions of interest are shown on full‐coverage susceptibility maps in a representative healthy volunteer and the numerical phantom. The white matter region of the healthy volunteer (posterior corona radiata) is not shown as it is not in this slice. (B) The coverage necessary for <5% decrease (Cov5%) in the susceptibility of the globus pallidus is shown in each healthy volunteer Figure S3 Susceptibility over varying slice thickness for different susceptibility calculation methods. Mean susceptibilities in 5 ROIs are plotted against slice thickness in a representative volunteer (A and D) and the numerical phantom (E and H) calculated using TKD (A, B, E, and F) or MEDI (C, D, G, and H). Note that here (unlike in Figures 5 and 6), the error bars in all graphs are equal to the SD within the ROIs. Axial slices of susceptibility maps from images at different slice thicknesses (B, C, F, and G) are also shown Figure S4 Susceptibility over varying coverage for different susceptibility calculation methods. Mean susceptibilities in 5 ROIs are plotted against coverage in a representative volunteer (A and D) and the numerical phantom (E and H) calculated using TKD (A, B, E, and F) or MEDI (C, D, G, and H). Note that here (unlike in Figures 5 and 6), the error bars in all graphs are equal to the SD in the ROIs. Axial slices of susceptibility maps from images at different FOVs (B, C, F, and G) are also shown [file MRM-81-1833-s001.pdf]

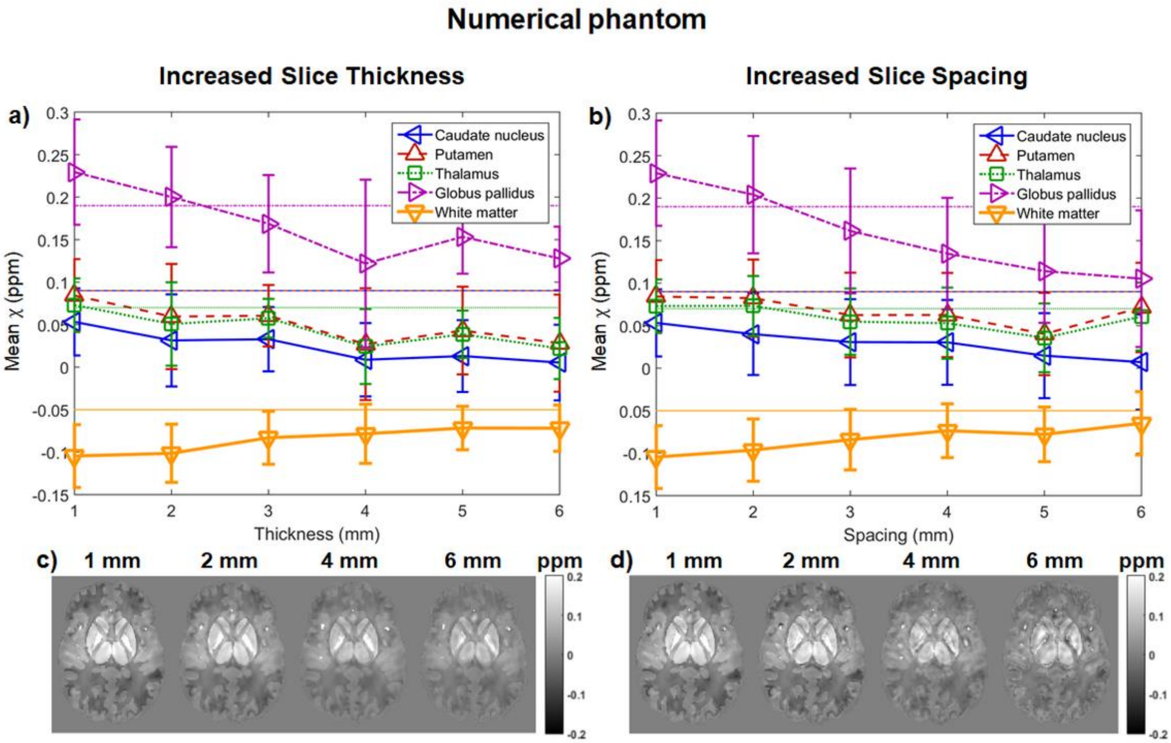

Figure S1: Susceptibility over varying slice thickness and slice spacing in the numerical phantom. Mean susceptibilities in five ROIs are plotted against slice thickness (a) and slice spacing (b). Horizontal lines indicate the corresponding ground-truth susceptibility values in both graphs. The data acquired have error bars equal to the standard deviation within the ROIs. Axial slices of susceptibility maps calculated from images at different slice thicknesses (c) and slice spacings (d) are also shown.

a) Regions of interest (ROIs)

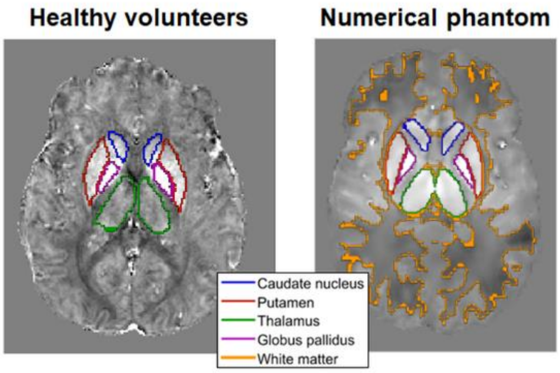

b) Coverage necessary for less than 5% error in the mean GP χ value

Size of GP in z direction as a % of the full FOV	Coverage (as % of the full FOV) corresponding to 5% error in χ	Coverage/Size ($Cov_{5\%}$)
9	41.5	4.6
9.2	30	3.3
9.5	40	4.2
8.8	34.5	3.9
10.1	50	4.9
**Mean ± Standard deviation =**		**4.2 ± 0.6**

Figure S2: (a) Regions of interest are shown on full-coverage susceptibility maps in a representative healthy volunteer and the numerical phantom. The white matter region of the healthy volunteer (posterior corona radiata) is not shown as it is not in this slice. (b) The coverage necessary for less than 5% decrease ($Cov_{5\%}$) in the susceptibility of the globus pallidus is shown in each healthy volunteer.

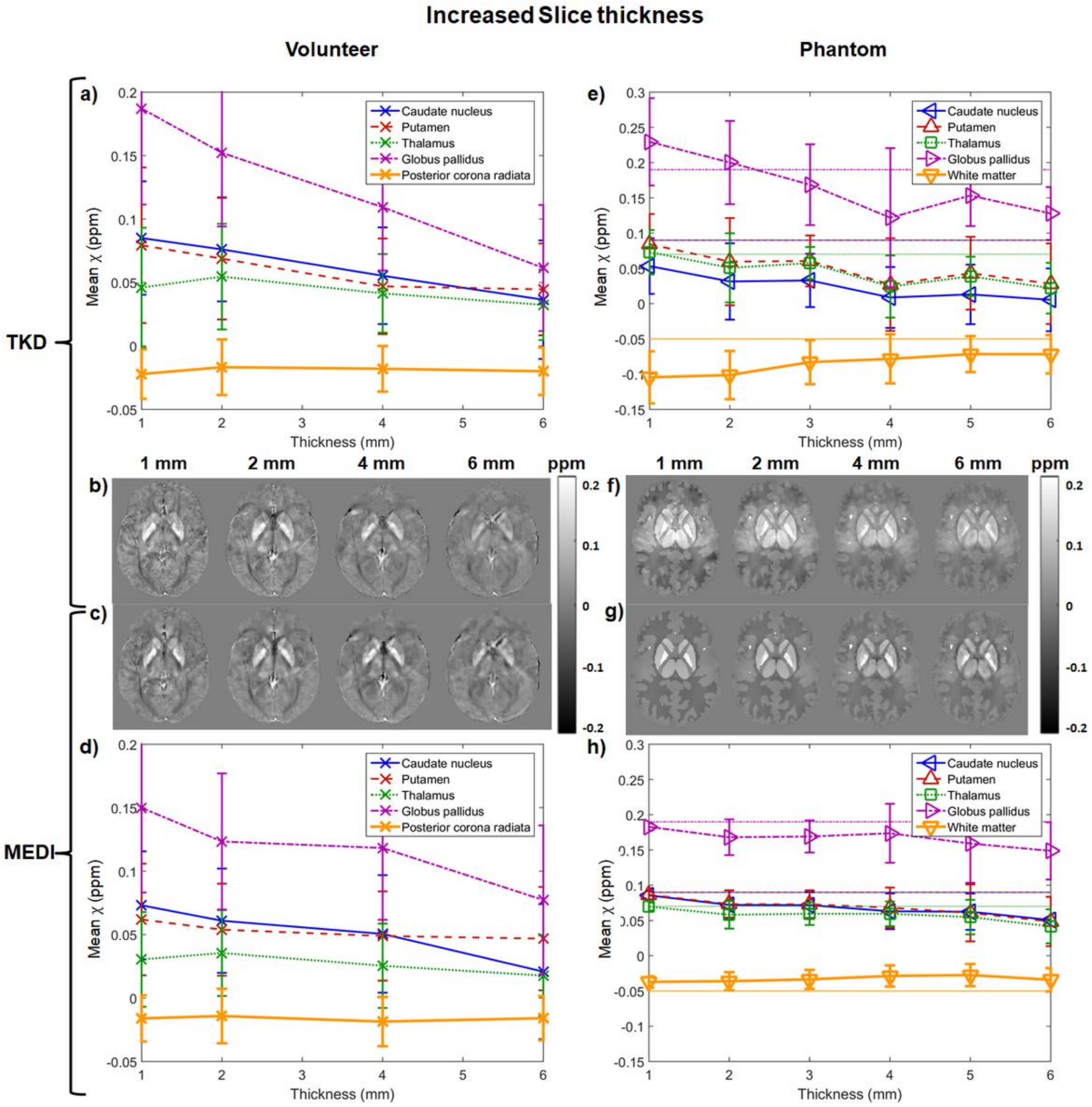

Figure S3: Susceptibility over varying slice thickness for different susceptibility calculation methods. Mean susceptibilities in five ROIs are plotted against slice thickness in a representative volunteer (a, d) and the numerical phantom (e, h) calculated using TKD (a-b, e-f) or MEDI (c-d, g-h). Note that here (unlike in Figures 5 and 6), the error bars in all graphs are equal to the standard deviation within the ROIs. Axial slices of susceptibility maps from images at different slice thicknesses (b-c, f-g) are also shown.

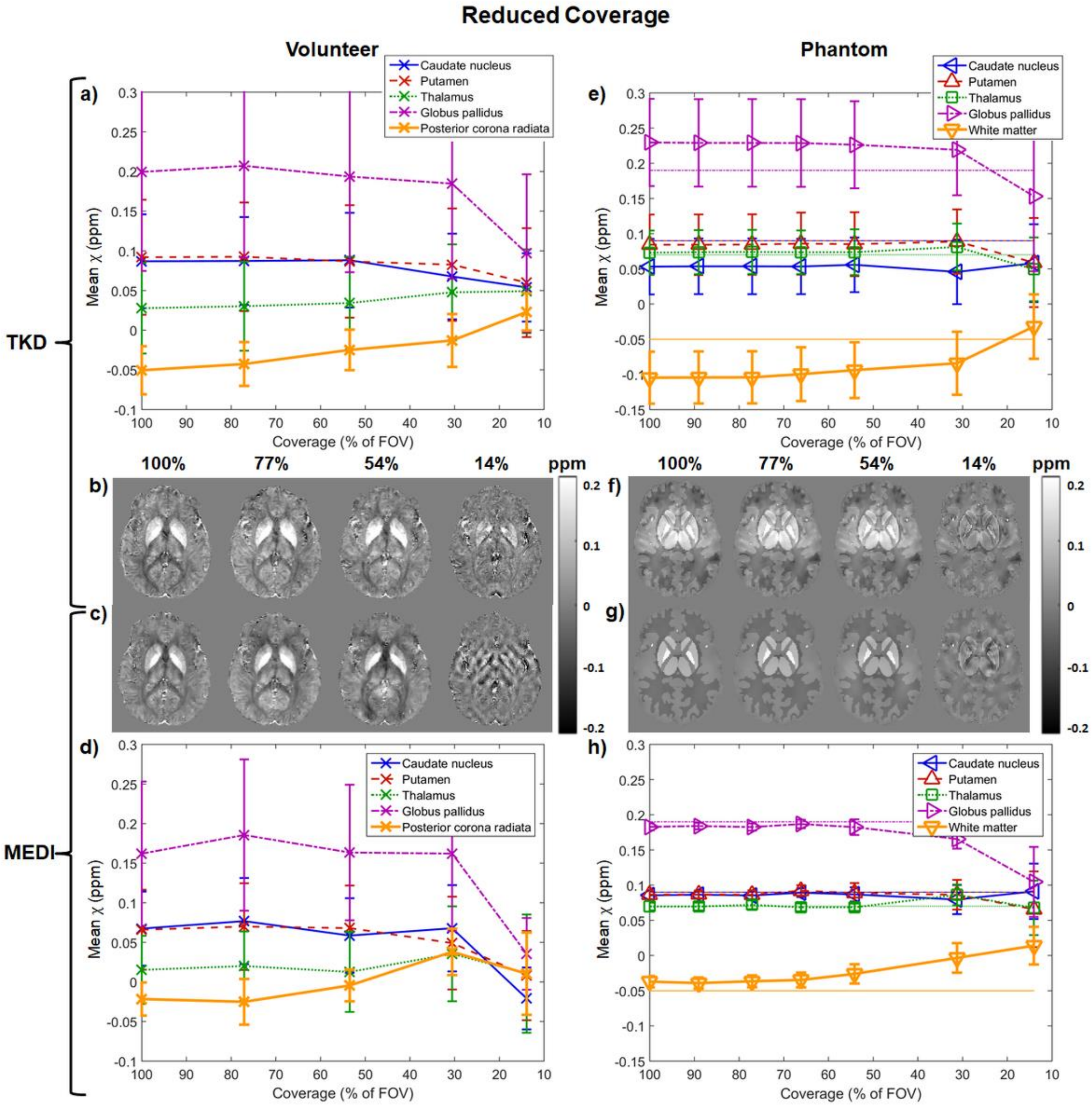

Figure S4: Susceptibility over varying coverage for different susceptibility calculation methods. Mean susceptibilities in five ROIs are plotted against coverage in a representative volunteer (a, d) and the numerical phantom (e, h) calculated using TKD (a-b, e-f) or MEDI (c-d, g-h). Note that here (unlike in Figures 5 and 6), the error bars in all graphs are equal to the standard deviation in the ROIs. Axial slices of susceptibility maps from images at different FOVs (b-c, f-g) are also shown.
